# Direct Association of Heat Shock Protein 20 (HSPB6) with Phosphoinositide 3-kinase (PI3K) in Human Hepatocellular Carcinoma: Regulation of the PI3K Activity

**DOI:** 10.1371/journal.pone.0078440

**Published:** 2013-11-06

**Authors:** Rie Matsushima-Nishiwaki, Takashi Kumada, Tomoaki Nagasawa, Mariko Suzuki, Eisuke Yasuda, Seiji Okuda, Atsuyuki Maeda, Yuji Kaneoka, Hidenori Toyoda, Osamu Kozawa

**Affiliations:** 1 Department of Pharmacology, Gifu University Graduate School of Medicine, Gifu, Japan; 2 Department of Gastroenterology, Ogaki Municipal Hospital, Ogaki, Gifu, Japan; 3 Department of Radiological Technology, Suzuka University of Medical Science, Suzuka, Mie, Japan; 4 Department of Medical Technology, Ogaki Municipal Hospital, Ogaki, Gifu, Japan; 5 Department of Surgery, Ogaki Municipal Hospital, Ogaki, Gifu, Japan; The University of Hong Kong, China

## Abstract

HSP20 (HSPB6), one of small heat shock proteins (HSPs), is constitutively expressed in various tissues and has several functions. We previously reported that the expression levels of HSP20 in human hepatocellular carcinoma (HCC) cells inversely correlated with the progression of HCC, and that HSP20 suppresses the growth of HCC cells via the AKT and mitogen-activated protein kinase signaling pathways. However, the exact mechanism underlying the effect of HSP20 on the regulation of these signaling pathways remains to be elucidated. To clarify the details of this effect in HCC, we explored the direct targets of HSP20 in HCC using human HCC-derived HuH7 cells with HSP20 overexpression. HSP20 proteins in the HuH7 cells were coimmunoprecipitated with the p85 regulatory subunit and p110 catalytic subunit of phosphoinositide 3-kinase (PI3K), an upstream kinase of AKT. Although HSP20 overexpression in HCC cells failed to affect the expression levels of PI3K, the activity of PI3K in the unstimulated cells and even in the transforming growth factor-α stimulated cells were downregulated by HSP20 overexpression. The association of HSP20 with PI3K was also observed in human HCC tissues *in vivo*. These findings strongly suggest that HSP20 directly associates with PI3K and suppresses its activity in HCC, resulting in the inhibition of the AKT pathway, and subsequently decreasing the growth of HCC.

## Introduction

Heat shock proteins (HSPs) are induced by a variety of stresses, such as heat and chemical stress [1]. HSPs have recently been classified into seven groups, including HSPA (HSP70), HSPB (small HSPs), HSPC (HSP90) and HSPH (HSP110) [2], [3]. High-molecular-weight HSPs, such as HSPA (HSP70), HSPC (HSP90) and HSPH (HSP110), have been well characterized and are recognized to act as molecular chaperones which prevent the aggregation of unfolded proteins, giving them a cytoprotective function [1], [4]. On the other hand, the human genome also encodes at least 10 small HSPs [3], [5]. Small HSPs (HSPB) with monomeric molecular masses ranging from 15 to 30 kDa, such as HSP27 (HSPB1), αB-crystallin (HSPB5) and HSP20 (HSPB6) are constitutively expressed in cells and tissues such as skeletal, smooth and cardiac muscles. The HSPB family is currently considered to play essential roles, such as in protein intracellular transport and in protecting the cytoskeletal architecture [3]. The small HSPs have significant similarities in terms of their amino acid sequences, known as the α-crystallin domain [3], [6]. Among the ten known small HSPs, it has been shown that HSP20 (HSPB6) has particularly versatile functions, and is associated with processes ranging from insulin resistance, to the prevention of vasospasms, to airway smooth muscle relaxation, and also has been demonstrated to have a protective function in the heart [7]–[10]. We have previously shown that HSP20 acts extracellularly to inhibit the platelet aggregation induced by thrombin or botrocetin [11], [12]. However, the exact roles of HSP20 have not yet been fully clarified.

Human hepatocellular carcinoma (HCC) is the fifth most common cancer and is the third leading cause of cancer-related death worldwide. Even after resection of the primary tumor, recurrence is common, and the survival rate is 30–40% at five-year post-surgery [13]. There is accumulating evidence that the growth factor receptor signaling pathways are dysregulated in human HCC [14], [15]. Phosphoinositide 3-kinase (PI3K) phosphorylates phosphatidylinositol lipids in response to various growth factors. PI3K, which transmit signals from growth factor receptor tyrosine kinases (RTKs), consists of heterodimers of the p85 regulatory subunit and the p110 catalytic subunit [16]–[18]. The primary function of the p85 regulatory subunit is to bind, stabilize and inhibit the p110 catalytic subunit until RTK activation [19], [20]. The p110 catalytic subunit of activated PI3K converts the plasma membrane lipid phosphatidylinositol-4,5-bisphosphate (PIP2) to phosphatidylinositol-3,4,5- triphosphate (PIP3).

The p110 catalytic subunit is encoded by three genes, α, β and δ. The p110α and p110β isoforms are ubiquitously expressed, while p110δ is largely leukocyte-specific [21]. Phosphatase and tensin homologue (PTEN), a phosphatase that catalyzes the dephosphorylation of the 3 position of PIP3, plays an important role as a negative regulator of the PI3K-AKT pathway [16], [17]. Phosphoinositide-dependent kinase 1 (PDK1), which is associated with PIP3, stimulates the phosphorylation of AKT, resulting in its activation. The activation of the AKT pathway affects cell growth, the cell cycle, cell survival and cytoskeletal rearrangement [16], [18]. Constitutive PI3K-AKT activation reportedly induces cancers of the endometrium, thyroid, prostate, breast, intestine and liver [18].

Studies on the pathogenesis of HCC have identified mutations in PI3K [13], and knockdown of the p85 regulatory subunit of PI3K in the mouse liver activates AKT and induces aggressive and high-grade HCC [22]. In our previous study [23], [24], we reported that the HSP20 expression levels inversely correlate with the TNM stage of human HCC, and that the overexpression of HSP20 in human HCC-derived HuH7 cells represses cell proliferation. The activation of the AKT pathway and the mitogen-activated protein kinases pathways, including the extracellular signal-regulated kinase (ERK) and c-Jun N-terminal kinase pathways induced by either transforming growth factor-α (TGFα) or hepatocyte growth factor, are suppressed in HSP20-overexpressing cells [24]. However, the exact mechanisms behind the effects of HSP20 in HCC remain to be elucidated.

The aim of this study was to clarify the direct target of HSP20 involved in the inhibition of the AKT pathway in HCC. We herein demonstrate that HSP20 interacts with PI3K and downregulates its activity in HCC.

## Materials and Methods

### Antibodies, Chemicals and Plasmids

HSP20 antibodies were purchased from Enzo Life Sciences Inc. (Farmingdale, NY, USA) and EMD Millipore Corp. (Billerica, MA, USA). Antibodies against PI3K p85, PI3K p110α, PI3K p110β, ERK (p44/p42 mitogen-activated protein kinase), MEK, Ras, AKT, phospho-AKT (Ser-473), phospho-AKT (Thr-308), PTEN and rabbit-IgG (peroxidase-conjugated, conformation specific) were purchased from Cell Signaling Technology, Inc. (Danvers, MA, USA). Glyceraldehyde-3-phosphate dehydrogenase (GAPDH) antibodies and rabbit IgG were purchased from Santa Cruz Biotechnology Inc. (Santa Cruz, CA, USA). Wild-type human HSP20 cDNA (clone ID 6074542), which was obtained from Open Biosystems, Inc. (Huntsville, AL, USA), was subcloned into the eukaryotic expression vector, pcDNA 3.1(+), as described previously [24]. The eukaryotic expression vector, pcDNA 3.1(+), Dynabeads protein A and Trizol reagent were purchased from Life Technologies Corp. (Carlsbad, CA, USA). Recombinant human TGFα was obtained from R&D systems Inc. (Minneapolis, MN, USA). LY294002 was purchased from ENZO Life Sciences Inc. The Omniscript Reverse Transcriptase kit was purchased from QIAGEN (Hilden, Germany). Fast-start DNA Master SYBR Green I was purchased from Roche Diagnostics K.K. (Basel, Switzerland). The BCA protein assay kit was obtained from Thermo Fisher Scientific Inc. (Waltham, MA, USA). The PI3K activity enzyme-linked immunosorbent assay (ELISA) kit (PI3-Kinase Activity ELISA: Pico) was purchased from Echelon Biosciences Inc., (Salt Lake City, UT, USA). All other materials and chemicals were obtained from commercial sources.

### Cell Culture and Transient Transfections

Human HCC-derived HuH7 cells were obtained from the Health Science Research Resources Bank (Tokyo, Japan). The HuH7 cells were maintained in Roswell Park Memorial Institute (RPMI) 1640 (Sigma-Aldrich Corp., St. Louis, MO, USA) medium supplemented with 1% fetal calf serum (FCS) (Hyclone Corp., Logan, UT, USA). For transfections, the HuH7 cells were cultured in 90 mm diameter dishes (1×10^6^ cells/dish) and transfected with 4 µg of the wild-type HSP20 plasmid or control (empty) pcDNA3.1(+) vector using the UniFector transfection reagent (B-Bridge International, Mountain View, CA, USA) in 4 ml of RPMI1640 medium without FCS. One day after transfection, the medium was changed to 6 ml of RPMI1640 medium with 1% FCS for coimmunoprecipitation and real-time RT-PCR experiments, or without FCS for the PI3K activity assay. The cells were then cultured for another 24 h.

### Protein Preparation

For coimmunoprecipitation, the transfected cells and snap-frozen HCC tissues were lysed in ice-cold TNE lysis buffer (10 mM Tris-HCl, pH 7.8, 1% Nonidet P-40, 150 mM NaCl, 1 mM EDTA, 1 mM dithiothreitol, 1 mM sodium fluoride, 1 mM sodium vanadate and protease inhibitor cocktail (Roche Diagnostics K.K.)). The lysates were then centrifuged at 10,000×*g* at 4°C for 30 min, and the supernatant was collected as TNE-soluble proteins. For the Western blot analysis of AKT and phospho-AKT, the transfected cells were pretreated with the indicated concentrations of LY294002 or vehicle for 60 min, and then stimulated with 20 ng/ml TGFα or vehicle for 10 min. After stimulation, the cells were lysed, homogenized and sonicated in lysis buffer, as described previously [23], [24].

### Coimmunoprecipitation

The indicated antibodies were added to the TNE-solubilized proteins, and the mixture was shaken gently overnight at 4°C, followed by the addition of 50 µl of Dynabeads protein A and incubation for a further 1 h with continuous mixing. Protein immunocomplexes were isolated with the use of a magnetic particle concentrator (6-tube magnetic separation rack, New England BioLabs Inc., Ipswich, MA, USA). The immunoprecipitated proteins and TNE-soluble proteins (for analysis total protein) were resuspended in the loading buffer for sodium dodecyl sulfate (SDS)-polyacrylamide gel electrophoresis (PAGE), heated at 95°C for 5 min, and analyzed by a Western blot analysis using peroxidase-labeled rabbit IgG (conformation specific) (L27A9) monoclonal antibodies (Cell Signaling Technology, Inc.).

### Western Blot Analysis

A Western blot analysis was performed as described previously [23]. Briefly, SDS-PAGE was performed by the method described by Laemmli [25]. The proteins in the gel were transferred onto polyvinylidene fluoride (PVDF) membranes, which were then blocked with 5% fat-free dry milk in phosphate-buffered saline (PBS) with 0.1% Tween20 for 1 h before incubation with the indicated primary antibodies. Peroxidase-labeled rabbit IgG antibodies were used as secondary antibodies. The peroxidase activity on the PVDF membranes was visualized on X-ray film by means of an ECL Western blotting detection system (GE Healthcare, Waukesha, WI, USA) as described in the manufacturer’s protocol.

### PI3K Activity Assay

The cultured HSP20-overexpressing cells were stimulated with or without 20 ng/ml TGFα for 10 min. After stimulation, the PI3K activity in the cells was determined using a PI3-Kinase Activity ELISA kit according to the manufacturer’s instructions. The absorbance of samples was measured at 450 nm with an EL 340 Bio Kinetic Reader (Bio-Tek Instruments, Inc., Winooski, VT, USA).

### Real-time RT-PCR

Total RNA was isolated and transcribed into complementary DNA using the Trizol reagent and Omniscript Reverse Transcriptase Kit, respectively. Real-time RT-PCR was performed using a Light Cycler system (Roche Diagnostics) in capillaries with the Fast-Start DNA Master SYBR Green I provided with the kit. Sense and antisense primers were synthesized based on the report by Biéche *et al.* for human PI3KR1 mRNA [26]. The sense and antisense primers for human GAPDH mRNA were purchased from Takara Bio Inc. (Tokyo, Japan) (primer set ID:HA067812). The PI3KR1 mRNA levels were normalized to those of GAPDH mRNA.

### Tissue Specimens

HCC tissues were obtained by surgical resection from patients in the Department of Surgery, Ogaki Municipal Hospital (Gifu, Japan) according to a protocol approved by the committee for the conduct of human research at Ogaki Municipal Hospital and at Gifu University Graduate School of Medicine. Written informed consent was obtained from all of the patients.

### Statistical Analysis

The data are expressed as the means ± SD. The statistical significance of the data from the cell culture experiments was analyzed using the Mann-Whitney U test. All *P* values were derived from two-tailed tests, and values of *P*<0.05 were considered to statistically significant. Each experiment was repeated three times with similar results.

## Results

### HSP20 does not Directly Interact with AKT or ERK in HCC Cells

In our previous study [23], [24], we showed that HSP20 is expressed in the tumor tissues of human HCC. However, HCC cell lines do not express the HSP20 protein. Therefore, we transfected wild-type HSP20 cDNA into HuH7 cells, a HCC-derived cell line, to make them express the HSP20 protein, and then analyzed its function.

**Figure 1:**
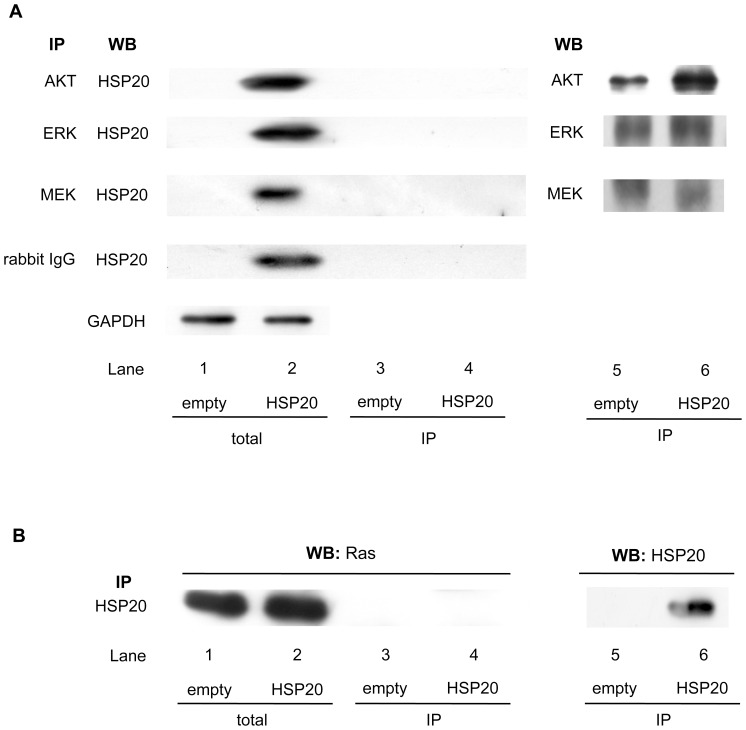
ERK, MEK, AKT and Ras do not directly interact with HSP20. (**A**) The expressions of HSP20 and GAPDH in the pre-immunoprecipitated cell lysates of control empty vector (lanes 1) or HSP20-overexpressing (lanes 2) HuH7 cells were determined by a Western blot analysis (total). The empty vector and HSP20-overexpressing HuH7 cell lysates were immunoprecipitated (IP) with antibodies for AKT, ERK, MEK and normal rabbit IgG followed by Western blotting (WB) using HSP20 antibodies (lanes 3 and 4). Immunoprecipitation of AKT, ERK and MEK proteins in the HuH7 cells transfected with empty vector or wild-type HSP20 vector was confirmed by WB using AKT antibodies, ERK antibodies and MEK antibodies, respectively (lanes 5 and 6). (**B**) The expression of Ras in the pre-immunoprecipitated cell lysates of control empty vector transfected (lane 1) or HSP20-overexpressing (lane 2) HuH7 cells were determined by a Western blot analysis (total). The empty vector transfected or HSP20-overexpressing HuH7 cell lysates were immunoprecipitated (IP) with HSP20 antibodies followed by Western blotting (WB) using Ras antibodies (lanes 3 and 4). Immunoprecipitation of HSP20 proteins in the HuH7 cells transfected with empty vector or wild-type HSP20 vector was confirmed by WB using HSP20 antibodies (lanes 5 and 6).

We also previously demonstrated, that HSP20 inhibits the activation of AKT and ERK via MEK, the upstream kinase of ERK, in human HCC tissues [24]. In cardiomyocytes, HSP20 reportedly interacts with phosphorylated AKT and maintains it in its phosphorylated state [27]. Therefore, we first investigated whether HSP20 directly interacts with these signal molecules, AKT, ERK and/or MEK, in the HuH7 cells. Although HSP20 was overexpressed in the transfected HuH7 cells (Figure 1A, lane 2 in comparison with lane 1), it was not coimmunoprecipitated with AKT, ERK or MEK (Figure 1A, lane 4).

We then examined whether HSP20 could interact with Ras, which is considered to function upstream of MEK [28]. Although the Ras protein was highly expressed in both the empty and HSP20 vector-transfected HuH7 cells (Figure 1B, lanes 1 and 2), it was not coimmunoprecipitated with HSP20 (Figure 1B, lane 4).

**Figure 2:**
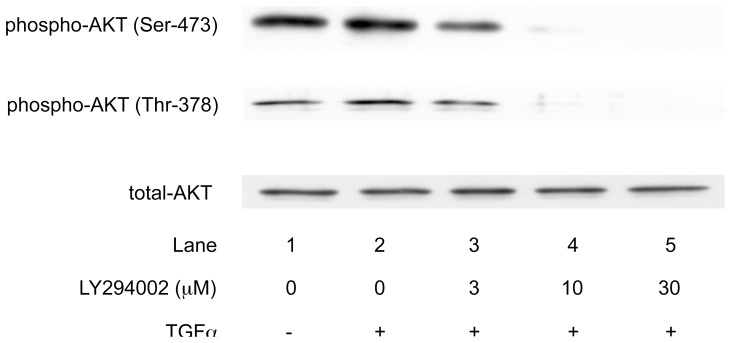
PI3K is an upstream kinase of AKT pathway in the HCC cells. HuH7 cells were treated with the indicated doses of LY294002, an inhibitor of PI3K, for 60/ml TGFα for 10 min. The activities of AKT were determined by the levels of phospho-AKT (Ser-473) and phospho-AKT (Thr-378) using a Western blot analysis.

It is generally recognized that PI3K is an upstream kinase of AKT [16], . We therefore confirmed that PI3K acts upstream of AKT in the HCC cells. A PI3K inhibitor, LY294002 [29], dose-dependently suppressed the TGFα-induced AKT phosphorylation at both the serine and threonine residues in the HuH7 cells, suggesting that PI3K also regulates the AKT activity in HCC cells (Figure 2).

### HSP20 Represses the PI3K Activity in HCC Cells

**Figure 3:**
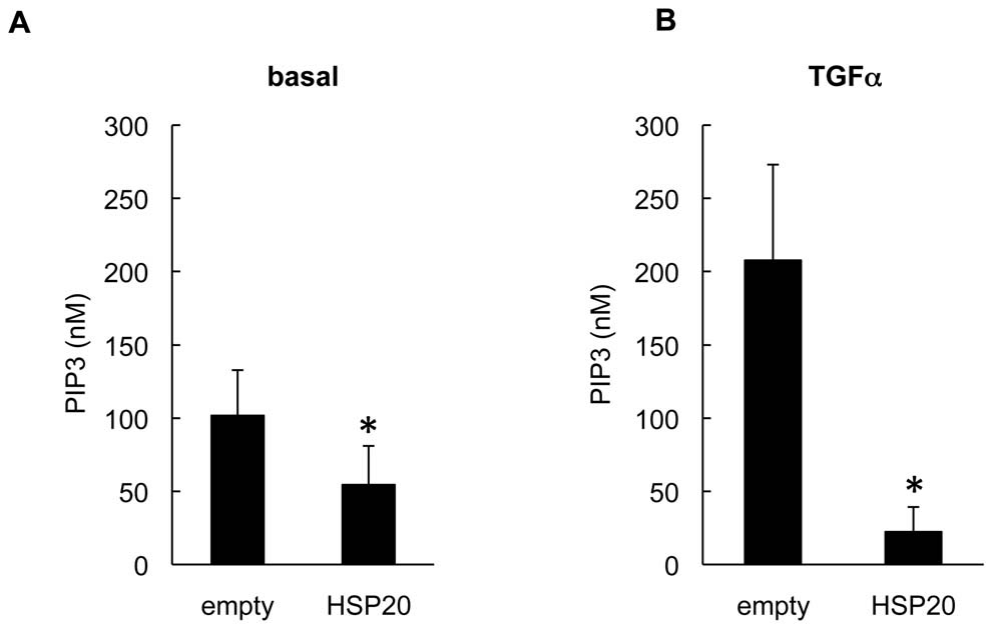
HSP20 represses the PI3K activities in the HCC cells. The HSP20-overexpressing HuH7 cells were stimulated with (**B**) or without (**A**) 20 ng/ml TGFα for 10 min. After stimulation, PI3K in the cells were immunoprecipitated followed by determination of the PIP3 producing activities. Values represent the means ± SD (n = 8). *: P<0.05.

Next we examined whether HSP20 affects the PI3K activity in the HuH7 cells. We found that the basal activity level of PI3K in the HSP20-overexpressing HuH7 cells was significantly repressed compared with that in the empty vector-transfected cells (Figure 3A). Accumulating evidence suggests that the protumorigenic growth factor signaling pathways, such as the TGFα/epidermal growth factor (EGF) signaling pathway, are dysregulated in human HCC [14], [15]. PI3K is activated by RTKs, including the receptor for TGFα [16], [18], [19]. We have previously reported that HSP20 inhibits the TGFα-stimulated AKT signaling pathway in HuH7 cells [24]. Therefore, we examined the PI3K activity in the HSP20-overexpressing HuH7 cell in the presence of TGFα stimulation. Compared with basal state, the PI3K activity in the empty vector-transfected HuH7 cells was enhanced by TGFα stimulation (Figure 3B, left column in comparison to Figure 3A, left column). The TGFα-induced PI3K activity was significantly repressed in the HSP20-overexpressing HuH7 cells (Figure 3B, right column).

### HSP20 does not Affect the Expression of PI3K or PTEN in HCC Cells

**Figure 4:**
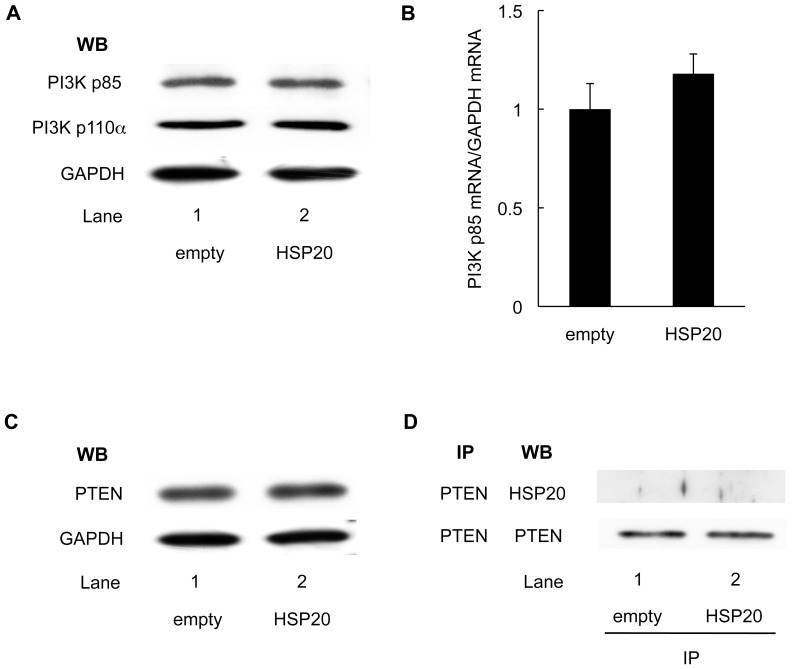
HSP20 does not affect the expression of PI3K and PTEN in HCC cells. (**A**) The protein levels of PI3K p85 and PI3K p110α subunits in empty vector transected (lane 1) and HSP20-overexpressing (lane 2) HuH7 cells were determined by a Western blot analysis using PI3K p85 antibodies and PI3K p110α antibodies. (**B**) The expressions of PI3K p85 mRNA and GAPDH mRNA were analyzed by real-time RT-PCR. The relative levels of PI3K p85 were normalized by GAPDH. The values are the means ± SD (n = 3). (**C**) The protein levels of PTEN in empty vector transfected (lane 1) and HSP20-overexpressing (lane 2) HuH7 cells were determined by a Western blot analysis using PTEN antibodies. (**D**) The empty vector transfected (lane 1) and HSP20-overexpressing (lane 2) HuH7 cell lysates were immunoprecipitated (IP) with PTEN antibodies, followed by Western blotting (WB) using HSP20 antibodies. Immunoprecipitation of PTEN proteins in the HuH7 cells transfected with empty vector or wild-type HSP20 vector was confirmed by WB using PTEN antibodies.

To clarify the effect of HSP20 on PI3K in HCC, we examined the protein levels of PI3K in the HSP20-overexpressing HuH7 cells. The levels of both PI3K p85 and PI3K p110α were not affected by HSP20 overexpression (Figure 4A). In addition, the expression level of PI3K p85 mRNA also did not show any significant difference in the cells with HSP20 overexpression compared with that in the control cells (Figure 4B). These findings suggest that HSP20 does not affect the synthesis or stability of PI3K in HCC cells.

PTEN, a tumor suppressor protein, acts as a lipid phosphatase on PIP3, and prevents AKT activation [16], [17]. We examined whether HSP20 affects PTEN in the HCC cells. As shown in Figure 4C, the protein levels of PTEN were not changed by HSP20 overexpression. Furthermore, the HSP20 in the HuH7 cells was not coimmunoprecipitated with PTEN (Figure 4D).

### HSP20 Directly Interacts with PI3K in HCC Cells

**Figure 5:**
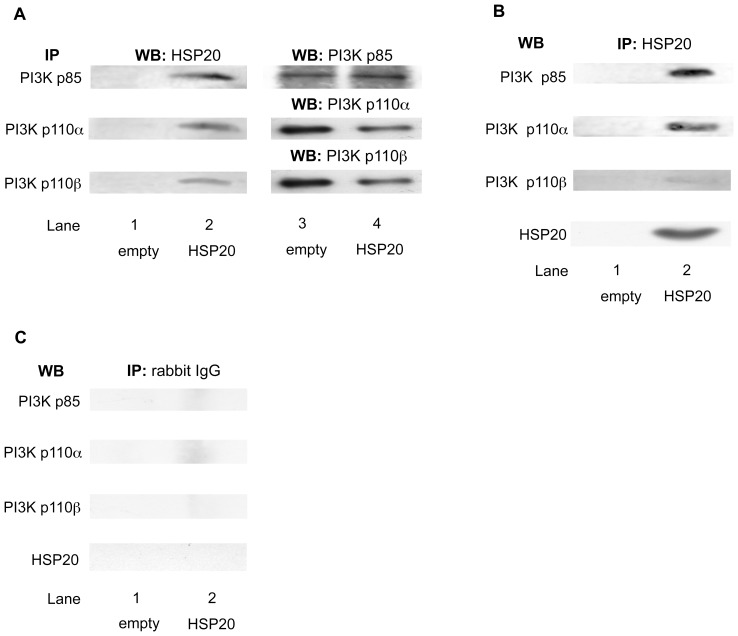
HSP20 directly interacts with PI3K in the HCC cells. (**A**) The empty vector transfected (lane 1) and HSP20-overexpressing (lane 2) HuH7 cell lysates were immunoprecipitated (IP) with antibodies for PI3K p85, PI3K p110α and PI3K p110β subunits, followed by Western blotting (WB) using HSP20 antibodies. Immunoprecipitation of the PI3K p85, PI3K p110α and PI3K p110β subunits in the HuH7 cells transfected with empty vector or wild-type HSP20 vector was confirmed by WB using PI3K p85 antibodies, PI3K p110α antibodies and PI3K p110β antibodies, respectively (lanes 3 and 4). (**B and C**) The empty vector transfected (lanes 1) and HSP20-overexpressing (lanes 2) HuH7 cell lysates were immunoprecipitated (IP) with antibodies for HSP20 (**B**) or normal rabbit IgG (**C**), followed by Western blotting (WB) using PI3K p85 antibodies, PI3K p110α antibodies, PI3K p110β antibodies or HSP20 antibodies.

Next, we examined whether HSP20 directly interacts with PI3K in HCC cells. As shown in Figure 5A, the HSP20 protein in the HSP20-overexpressing HuH7 cells was coimmunoprecipitated with PI3K p85, the regulatory subunit, and also with PI3K p110α and PI3K p110β, the catalytic subunits (Figure 5A, lane 2 in comparison with lane 1). In addition, HSP20 antibodies pulled down PI3K p85, PI3K p110α and PI3K p110β from the HSP20-overexpressing HuH7 extracts (Figure 5B, lane 2 in comparison with lane 1). We also demonstrated that the overexpressed HSP20 protein in the HuH7 cells was also immuoprecipitated by HSP20 antibodies (Figure 5B). Furthermore, we found that PI3K p85, PI3K p110α, PI3K p110β and HSP20 were not immunoprecipitated with normal rabbit IgG (Figure 5C). These results suggest that the HSP20 protein directly interacts with the PI3K protein in HCC cells.

### HSP20 also Interacts with PI3K in Human HCC Tissues

**Figure 6:**
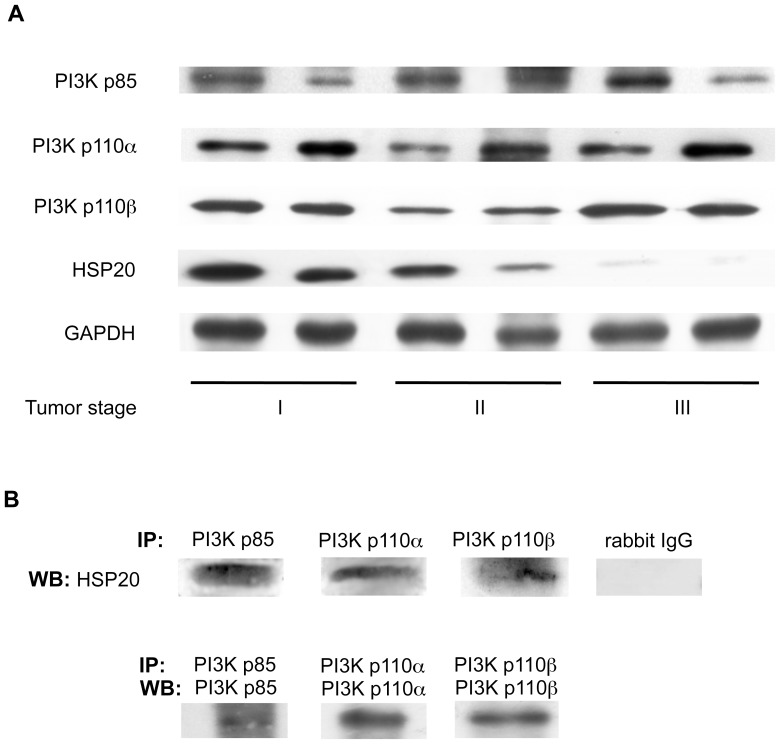
HSP20 interacts with PI3K in human HCC tissues. (**A**) The protein levels of HSP20 and PI3K p85, PI3K p110α and PI3K p110β subunits in stages I, II and III human HCC tissues were compared by a Western blot analysis. (**B**) The lysates from a stage II HCC tissue were immunoprecipitated (IP) with antibodies for PI3K p85, PI3K p110α PI3K p110β or normal rabbit IgG followed by Western blotting (WB) using HSP20 antibodies. Immunoprecipitation of the PI3K p85, PI3K p110α and PI3K p110β subunits in the stage II HCC tissue lysates was confirmed by WB using PI3K p85 antibodies, PI3K p110α antibodies and PI3K p110β antibodies, respectively.

Because stable cell lines do not always accurately reflect the clinical situation, we also examined the interaction between PI3K and HSP20 in human HCC tissue specimens. We have previously reported that the HSP20 expression levels are gradually decreased with tumor progression [23]. Although the HSP20 protein levels in the stage III HCC tissue samples were much lower than those in the stage I HCC tissues, as described previously, we found that the PI3K p85, PI3K p110α and PI3K p110β proteins are all expressed in stages I, II and III human HCC (Figure 6A). We also showed that the HSP20 in the HCC tissue samples was coimmunoprecipitated with PI3K p85, PI3K p110α and PI3K p110β suggesting that the HSP20 protein also directly interacts with the PI3K protein in human HCC tissues *in vivo* (Figure 6B).

## Discussion

HSP20 reportedly interacts with several cellular proteins, such as 14-3-3 and actin [30], [31]. We have previously shown that HSP20 inhibits the AKT signaling pathway in HCC cells [24]. In normal cardiomyocytes and mesenchymal stem cells, HSP20 has also been shown to interact with phosphorylated AKT and preserve its activity [27], [32]. In the present study, we first investigated the relationship between HSP20 and AKT in HCC. However, we were not able to observe a direct interaction between the HSP20 and AKT proteins in the HSP20-overexpressing HCC cells. It is possible that the effects of HSP20 on AKT might differ between normal cardiomyocytes or mesenchymal stem cells and HCC cells. The binding partner(s) of HSP20 and their interaction(s) might be dependent on the cell types.

Therefore, to explore how HSP20 regulates AKT signaling in HCC, we focused on the interaction of HSP20 with PI3K, an upstream kinase of AKT. We found that the PI3K activity was down-regulated in the HSP20-overexpressing HCC cells compared with the control cells. It has been reported that an increased level of the PI3K p85α regulatory subunit inhibits the PI3K activity [33]. However, the amounts of both the PI3K p85α and PI3K p110α subunits in the HSP20-overepressing HCC cells were similar to those in the control cells.

PTEN negatively regulates the AKT activity at a point upstream of AKT, and the loss of PTEN function leads to the constitutive activation of the PI3K-AKT pathway [17]. Therefore, we examined whether HSP20 affects and/or associates with PTEN, a phosphatase of PIP3, in the HSP20-overexpressing HCC cells. However, we were not able to find any effect of HSP20 on the PTEN protein expression, and no interaction between HSP20 and PTEN was observed. It therefore seems unlikely that HSP20 can regulate the activity of PI3K via the protein expression levels of either the PI3K or PTEN proteins.

In this study, we also showed that HSP20 associated with the PI3K p85 subunit and PI3K p110 subunit in the HSP20-overexpressing HCC cell line. Furthermore, we demonstrated that there was an interaction between HSP20 and PI3K in the clinical specimens from patients with HCC. Both the PI3K p85 subunit and PI3K p110 subunit were immunoprecipitated simultaneously by HSP20 antibodies. HSP20 might interact with PI3K p85/p110 dimers. It has been shown that the binding of phosphoproteins to the SH2 domains of PI3K p85 activates the PI3K p85/p110 dimers by inducing a transition from an inhibited to a disinhibited state [20]. Therefore, it is likely that the association of HSP20 with PI3K p85 and/or p110 dimers may prevent the interaction of phosphoproteins with the dimers and inhibit their activation, thus resulting in the suppression of HCC proliferation. There is a possibility that HSP20 directly binds to one of the subunits of PI3K dimers and that another subunit of the PI3K dimer is coimmunoprecipitated. In cases of HSP20 binding to the PI3K p110 subunit, it is likely that HSP20 directly suppresses PI3K activation in response to growth factors independently of the PI3K p85 subunit. On the other hand, when directly associated with the PI3K p85 subunit, HSP20 strengthens the regulatory role of the subunit. However, the precise molecular mechanisms underlying the regulation of PI3K by HSP20 in HCC cells remain to be elucidated.

It has been reported that PIK3A and PIK3R1, the genes that respectively encode the PI3K p110α and PI3K p85 subunits, are somatically mutated in many cancers, including liver cancer, and that these mutations promote the activation of the PI3K-AKT pathway and oncogenesis [34], [35]. Additionally, upregulated levels of the PI3K p110β subunit could induce oncogenic transformation [36]. Therefore, strict control of the PI3K activity is important for preventing oncogenesis. Based on our results, it is probable that the interaction of HSP20 with PI3K and its inhibition of the PI3K activity in HCC might play significant role in HCC development.

Currently, the PI3K pathway is considered to be an attractive target for therapeutic intervention in cancer. One PI3K inhibitor, an improved wortmannin analogue, PX-866, is currently being evaluated in clinical trials in patients with advanced solid tumors [37]. CAL101, a PI3K p110δ selective inhibitor, reportedly demonstrated a clinical benefit in patients with relapsed or refractory indolent non-Hodgkin’s lymphoma, mantle cell lymphoma and chronic lymphocytic leukemia [19]. These findings suggest that PI3K is indeed a valid target for cancer therapy. However, the details of HSP20 behind controlling PI3K activity are still not fully known and further investigation should be needed to elucidate the precise role of HSP20.

Although we have previously shown that HSP20 inhibits not only the AKT pathway, but also the ERK pathway [24], we were unable to find any interaction of HSP20 with ERK and MEK. It has been reported that the Ras protein acts at a point upstream of both PI3K and ERK [38]. However, we found that HSP20 did not associate with Ras in the HCC cells. PR-39, an endogenous antimicrobial peptide isolated from pig intestines and neutrophils, was reported to bind and inhibit PI3K, and it also decreased the MAP kinase activities and cyclin D1 expression [39]. It seems likely that the HSP20 in HCC might have an inhibitory effect on the activation of PI3K and MAP kinase, similar to PR-39. Further investigations are necessary to clarify the exact mechanism underlying how HSP20 affects the development and progression of HCC.

In conclusion, our findings strongly suggest that HSP20 directly associates with PI3K and regulates the PI3K-AKT activity in human HCC.
